# *CACNA1A* Mutations Causing Early Onset Ataxia: Profiling Clinical, Dysmorphic and Structural-Functional Findings

**DOI:** 10.3390/ijms22105180

**Published:** 2021-05-13

**Authors:** Antonio F. Martínez-Monseny, Albert Edo, Dídac Casas-Alba, Mercè Izquierdo-Serra, Mercè Bolasell, David Conejo, Loreto Martorell, Jordi Muchart, Laura Carrera, Carlos I. Ortez, Andrés Nascimento, Baldo Oliva, José M. Fernández-Fernández, Mercedes Serrano

**Affiliations:** 1 Department of Genetic and Molecular Medicine, Institut de Recerca, Hospital Sant Joan de Déu, 08950 Barcelona, Spain; afmartinez@sjdhospitalbarcelona.org (A.F.M.-M.); dcasasa@sjdhospitalbarcelona.org (D.C.-A.); mbolasell@sjdhospitalbarcelona.org (M.B.); lmartorell@sjdhospitalbarcelona.org (L.M.); 2 Laboratory of Molecular Physiology, Department of Experimental and Health Sciences, Universitat Pompeu Fabra, 08003 Barcelona, Spain; albert.edo@upf.edu (A.E.); merce.izquierdo@upf.edu (M.I.-S.); 3 Pediatric Department, Complejo Asistencial de Burgos, 09006 Burgos, Spain; dconejo@saludcastillayleon.es; 4 U-703 Centre for Biomedical Research on Rare Diseases (CIBER-ER), Instituto de Salud Carlos III, 08002 Barcelona, Spain; ciortez@sjdhospitalbarcelona.org (C.I.O.); anascimento@sjdhospitalbarcelona.org (A.N.); 5 Pediatric Radiology Department, Institut de Recerca Sant Joan de Déu, Hospital Sant Joan de Déu, 08950 Barcelona, Spain; jmuchart@hsjdbcn.org; 6 Neuropediatric Department, Institut de Recerca Sant Joan de Déu, Hospital Sant Joan de Déu, 08950 Barcelona, Spain; lcarrerag@sjdhospitalbarcelona.org; 7 Structural Bioinformatics Lab, Department of Experimental and Health Sciences, Universitat Pompeu Fabra, 08003 Barcelona, Spain; baldo.oliva@upf.edu

**Keywords:** ataxia, cerebellar atrophy, dysmorphic traits, early-onset cerebellar ataxia, *CACNA1A* gene, Ca_V_2.1 (P/Q-type) voltage-dependent calcium channel

## Abstract

The *CACNA1A* gene encodes the pore-forming α_1A_ subunit of the voltage-gated Ca_V_2.1 Ca^2+^ channel, essential in neurotransmission, especially in Purkinje cells. Mutations in *CACNA1A* result in great clinical heterogeneity with progressive symptoms, paroxysmal events or both. During infancy, clinical and neuroimaging findings may be unspecific, and no dysmorphic features have been reported. We present the clinical, radiological and evolutionary features of three patients with congenital ataxia, one of them carrying a new variant. We report the structural localization of variants and their expected functional consequences. There was an improvement in cerebellar syndrome over time despite a cerebellar atrophy progression, inconsistent response to acetazolamide and positive response to methylphenidate. The patients shared distinctive facial gestalt: oval face, prominent forehead, hypertelorism, downslanting palpebral fissures and narrow nasal bridge. The two α_1A_ affected residues are fully conserved throughout evolution and among the whole human Ca_V_ channel family. They contribute to the channel pore and the voltage sensor segment. According to structural data analysis and available functional characterization, they are expected to exert gain- (F1394L) and loss-of-function (R1664Q/R1669Q) effect, respectively. Among the *CACNA1A*-related phenotypes, our results suggest that non-progressive congenital ataxia is associated with developmental delay and dysmorphic features, constituting a recognizable syndromic neurodevelopmental disorder.

## 1. Introduction

The *CACNA1A* gene, located on chromosome 19p13, encodes the pore-forming α_1A_ subunit of the voltage-gated Ca_V_2.1 (P/Q-type) Ca^2+^ channel, which mediates the intracellular entry of Ca^2+^ ions [1]. *CACNA1A* plays a major role in neurotransmitter release throughout the nervous system, especially in cerebellar Purkinje cells and in all brain areas involved in the pathogenesis of migraine [1].

The Ca_V_2.1 (P/Q-type) Ca^2+^ channel is a high-voltage-activated channel consisting of a principal pore-forming subunit (α_1A_ or CACNA1A), associated with auxiliary subunits [2,3]. α_1A_ consists of four repeated homologous domains (DI–DIV), each with six transmembrane regions (S1-S6) that constitute two functional modules: a voltage sensor (S1-S4) and a Ca^2+^-selective pore (S5-P loop-S6) [2]. The movement of 5–6 positively charged residues located at the S4 helices (Arg^+^ or Lys^+^, every 3rd-4th amino acid (defined as R0-R5 gating charges)) induces the conformational changes required for pore opening in response to membrane depolarization [2].

Mutations in the *CACNA1A* gene result in great clinical heterogeneity, and present with chronic progressive symptoms or paroxysmal events (including sporadic and familial hemiplegic migraine (HM), epilepsy and migraine), or both [4]. Initially, mutations in the *CACNA1A* gene were identified in patients with familial hemiplegic migraine (FHM) (MIM#141500) or episodic ataxia type 2 (MIM#108500) [4], in that phenotypes acetazolamide may prevent recurrence and improve symptoms [5]. Triplet repeat mutations in the *CACNA1A* gene were associated with late onset-spinocerebellar ataxia type 6 (MIM#183086) characterized by late onset, slowly progressive cerebellar ataxia, due to toxic accumulation of an expanded polyQ [6]. Other mutations of *CACNA1A* were identified in patients with early onset epileptic encephalopathy (MIM#617106) [7]. Recently, single case reports and short case series have described patients with congenital (non-episodic) ataxia, with or without cerebellar atrophy, carrying missense *CACNA1A* mutations [8,9], located around pore regions, voltage-sensing regions and S4-S5 linkers connecting these two functional modules of Ca_V_2.1 [8]. Therefore, *CACNA1A* mutations should be included in the differential diagnosis of both congenital ataxia and early onset epileptic encephalopathy [7,8]. Unfortunately, clinical findings may be unspecific at early stages, radiology may show normal findings and dysmorphic features that could be of help have not yet been reported. To complete the phenotype, cognitive dysfunction, including learning difficulties and autism, have been reported among the chronic neurological manifestations associated with *CACNA1A* point-mutations [4,10,11].

We present the neurological and radiological features of three patients with congenital ataxia, two of them with a previously reported *CACNA1A* mutation and one carrying a new *CACNA1A* variant. We propose a pattern of dysmorphic facial features to facilitate diagnosis. Clinical and neuroradiological evolution, pharmacological management, structural localization of variants and their expected functional consequences are also depicted.

## 2. Results

### 2.1. Clinical Description Of Patients

**Figure 1:**
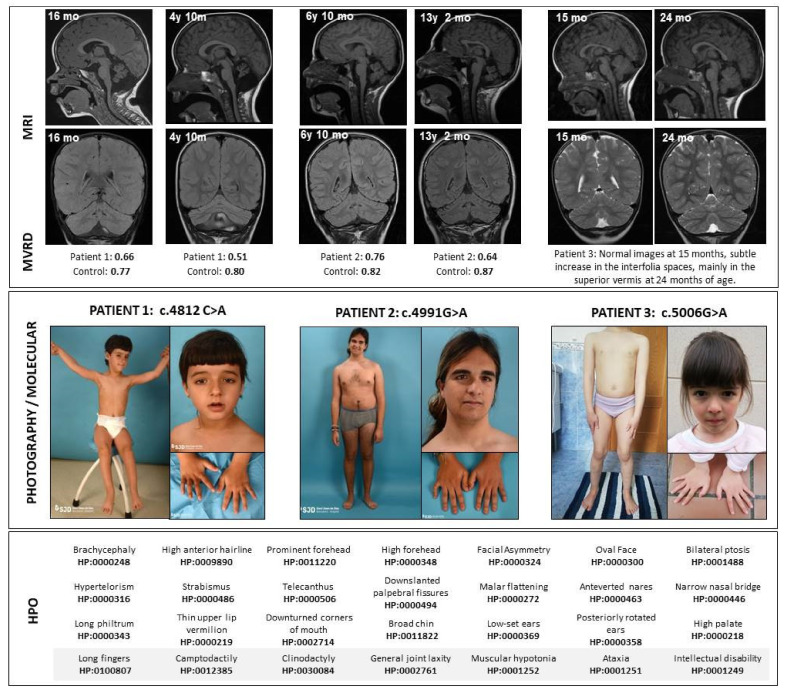
Clinical and radiological features of patients. Above, the magnetic resonance sagittal and coronal images show a progression in the cerebellar atrophy in Patients 1 and 2, despite clinical stabilization. Immediately below the images, the midsagittal vermis relative diameter (MVRD) has been calculated in the sagittal sequences for patients 1 and 2. MVRDs are detailed and compared to controls’ values. In the middle, pictures from the patients are shown. In the bottom, Human Phenotype Ontology (HPO) codes are included. y: years; mo: months; MVRD: midsagittal vermis relative diameter.

Patients’ molecular, clinical and radiological data are presented in Table 1 and Figure 1.

Pregnancies were uneventful; all the patients showed developmental delay, with particularly marked speech delay in Patient 1. They all presented persistent cerebellar symptoms in different degrees of severity: global hypotonia, truncal and limb ataxia, dysarthria or slurred speech and oculomotor symptoms such as nystagmus, oculomotor apraxia and strabismus. Patients 2 and 3 walk independently and are able to maintain a conversation at 20 years and 7 years of age, respectively. Patient 1, the most severely affected, is able to walk only with a walking frame and has severe communication impairment at 8 years of age. They have not shown neurological regression at any time. 

Acetazolamide was initiated in all three patients, applying a compassionate use formula, and maintained for at least 8 months. Responses are detailed in Table 1. In the case of Patient 2, at the age of 17 years he came into the emergency room because of subtle uncontrolled lateral head movements without consciousness abnormalities. A video EEG register during the episode ruled out epileptic activity, and the initiation of acetazolamide limited the episodes and improved the motor symptoms (three points in the ICARS). However, after 12 months of therapy he presented kidney lithiasis and the treatment was stopped. Subtle worsening in motor abilities was evident but no new abnormal movements appeared after the withdrawal.

The two younger patients showed abnormal executive functions and fulfilled attention deficit with hyperactivity disorder (ADHD), with positive response to guanfacine and methylphenidate, respectively. 

The three patients show common dysmorphic traits (Figure 1) such as a mildly asymmetric and oval face with large and prominent forehead, mild bilateral ptosis, strabismus, hypertelorism, telecanthus, downslanting palpebral fissures and narrow nasal bridge. Patients 1 and 2 also show low-set ears. In the midface, patients 1 and 3 show a long philtrum and a high palate. They all present joint laxity, but also long fingers, with clinodactyly of fifth fingers combined with marked camptodactyly of fifth fingers in the oldest patient.

Concerning neuroimaging, cranial MRI characteristics are detailed in Table 1, and images are included in Figure 1. In sequential MRI studies a progression in the cerebellar atrophy is marked, with unspecific or no findings in the supratentorial structures. Their MVRD was compared with two sex- and age-matched controls. 

### 2.2. Molecular Characterization of Patients

**Figure 2:**
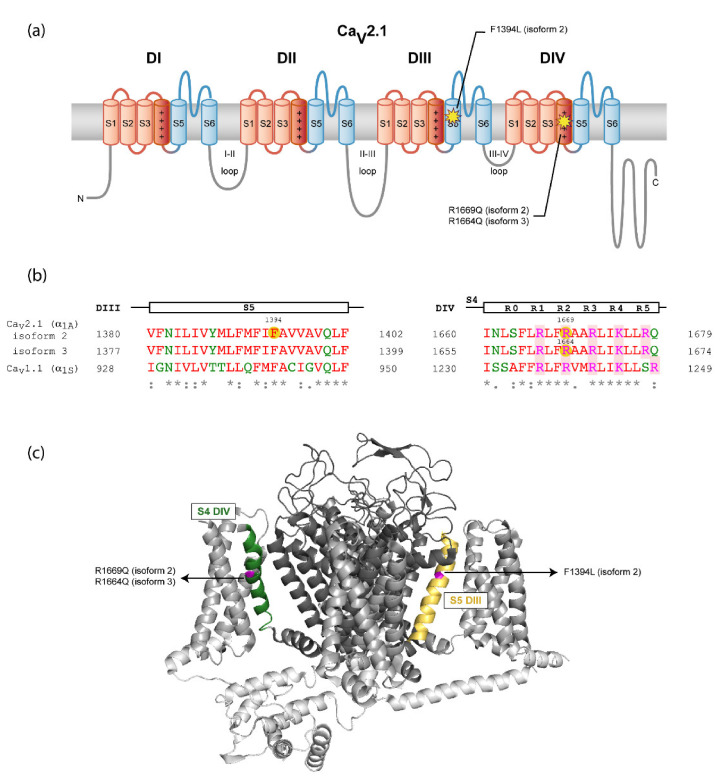
Location of the mutations in Ca_V_2.1 α_1A_ channel subunit. (**a**) Location of the variant residues of α_1A_ channel subunit isoforms 2 and 3 in the secondary structure of the protein according to the cryo-electron microscopy (cryo-EM) structure of the rabbit Ca_V_1.1 complex, containing the pore-forming α_1S_ and several regulatory subunits [12] (see also panel B). N- and C-termini and the intracellular loops are shown in gray, voltage-sensor modules (S1–S4) from the four domains are in red—with the S4 α-helixes colored in dark red—and Ca^2+^-selective pore modules (S5-P loop-S6) are in blue. (**b**) Sequence alignment of the regions affected by variants between the human α_1A_ subunit isoforms 2 and 3 and rabbit Ca_V_1.1 α_1S_ subunit. The α_1A_ subunit segment of each alignment is indicated at the top. Mutations are highlighted with yellow circles on the human Ca_V_2.1 (hCa_V_2.1) sequences. The gating charged residues of S4 segments (labeled R0-R5) are shaded in red. Amino acids are colored depending on their physicochemical properties: small and hydrophobic are in red, acidic in blue, basic in magenta and G and amino acids containing hydroxyl, sulfhydryl or amine groups in green. Below, a consensus code indicates fully conserved residues (*), conservation between residues with strongly similar properties (:) or with weakly similar properties (.). The Uniport IDs of the sequences aligned are for hCa_V_2 (α_1A_): O00555-2 (isoform 2) and O00555-3 (isoform 3) and for rabbit Ca_V_1.1 (α_1S_): P07293. The sequence alignments were made using Multiple Sequence Aligment Clustal Omega. (**c**) Three-dimensional location of the amino acid variants on a Ca_V_ channel model. The structure of the α_1S_ subunit of the Ca_V_1.1 channel (PDB 5GJV) [12] was used as a model considering their high level of homology (panel B). N- and C-termini and the intracellular loops are shown in light gray, voltage-sensor modules (S1–S4) are in gray and Ca^2+^-selective pore modules (S5-P loop-S6) are in dark gray. The two regions where mutations are located are highlighted in yellow for S5 helix of DIII and green for S4 helix of DIV. The residues of Ca_V_1.1 (α_1S_) equivalent to those mutated in Ca_V_2.1 (α_1A_) and identified in the patients, according to the sequence alignment on panel B, have been highlighted in magenta.

**Figure 3:**
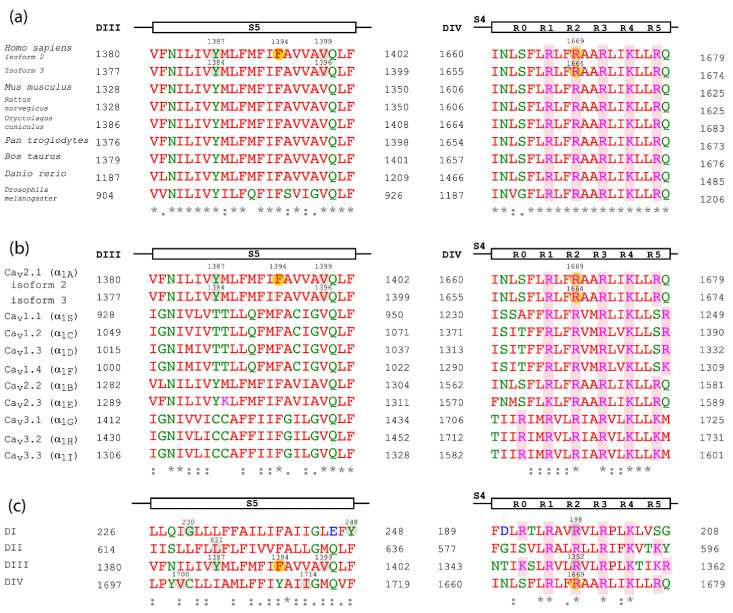
Sequence alignment of the α_1A_ subunit regions affected by the amino acid variants. Sequence alignment of the α_1A_ subunit regions affected by mutations, including α_1A_ isoforms 2 and 3, between different species (**a**), between the human Ca_V_ channel family (**b**) and between the four domains of α_1A_ subunit isoform 2 (**c**). The α_1A_ region of each alignment is indicated at the top. Ca_V_2.1 mutations are highlighted with yellow circles on the α_1A_ subunit isoform 2 or 3. Other Ca_V_2.1 residues affected by mutations mentioned in the text are indicated in gray circles. The gating charged amino acids of S4 segments (labeled R0-R5) and shaded in red. Residues are colored given their physicochemical properties: small and hydrophobic are in red, acidic in blue, basic in magenta and G and residues containing hydroxyl, sulfhydryl or amine groups in green. The consensus code below indicates fully conserved residues (*), conservative (:) or semi-conservative amino acid substitutions (.). The Uniport IDs of the aligned orthologous Ca_V_2.1 sequences are: *Mus musculus*: P97445; *Rattus norvegicus*: P54282; *Oryctolagus cuniculus*: P27884; *Pan troglodytes*: A0A2I3T217; *Bos taurus*: F1N1E0; *Danio rerio*: E9QJF6; *Drosophila melanogaster*: P91645. The IDs of the human Ca_V_ channel family aligned are the following hCa_v_1.1: Q13698; hCa_V_1.2: Q13936; hCa_V_1.3: Q01668; hCa_V_1.4: O60840; hCa_V_2.2: Q00975; hCa_V_2.3: Q15878; hCa_V_3.1: O43497; hCa_V_3.2: O95180; hCa_V_3.3: Q9P0X4. The sequence alignments were made using Multiple Sequence Aligment Clustal Omega. Here, residue numbers are indicated according to their position in α_1A_ subunit isoform 2 and 3; however, some of them differ from the nomenclature of the mutations found in the literature: R1350Q/R1349Q corresponds to R1352Q in isoform 2, Y1385C to Y1387C in isoform 2 and Y1384C in isoform 3, V1396M is V1399M in isoform 2 and V1695I is V1700I in isoform 2.

Regarding molecular findings, patient 1 had a likely pathogenic heterozygous missense mutation c.4182C>A (p.Phe1394Leu, F1394L) in the *CACNA1A* gene encoding α_1A_ isoform 2, NM_023035.2. Patient 2 and 3 had a similar likely pathogenic heterozygous missense mutation with different nomenclature due to isolation from two different *CACNA1A* genes encoding α_1A_ isoforms: c.4991G>A (p.Arg1664Gln, R1664Q) in the isoform 3 (NM_001127221.1) and c.5006G>A (p.Arg1669Gln, R1669Q) in the isoform 2 (NM_023035.2), respectively (Figure 2b and Figure 3, right).

By using the structure of the rabbit Ca_V_1.1 complex [12], the localizations of Ca_V_2.1 mutations can be predicted given their high level of homology. Thus, F1394L is located at the S5 segment of α_1A_ domain III (DIII), a few amino acids downstream from the beginning of the pore-loop between S5 and S6 helices. R1664Q and R1669Q are the same mutation in α_1A_ isoforms 3 and 2, respectively, and they affect the R2 gating charge at the voltage sensor S4 segment of domain IV (DIV) (Figure 2). The two affected residues, F1394 and R1664/R1669, are fully conserved throughout evolution (Figure 3a for comparison of orthologous Ca_V_2.1 channels) and among all the human Ca_V_ channel family [4] (Figure 3b). Supplementary Table S1 encompasses previously *CACNA1A* variants linked to congenital ataxia.

## 3. Discussion

We present three patients with non-progressive congenital ataxia without HM or epilepsy and common dysmorphic traits. Facial dysmorphic features of all patients include a mildly asymmetric and oval face with large and prominent forehead, mild bilateral ptosis, strabismus, hypertelorism, telecanthus, downslanting palpebral fissures and narrow nasal bridge. We have identified only one previous article describing dysmorphic traits in a patient harboring a pathogenic variant in *CACNA1A* [13]. She had congenital ataxia with severe HM and dysmorphic facial features, including round face with high forehead and an OFC in the 90–97th centile, and carried a ΔF1502 gain-of-function variant (located at the inner pore vestibule of the Ca_V_2.1 channel) [13]. Genetic diseases presenting congenital ataxia are frequently difficult to diagnose as cerebellar atrophy on the MRI may not be present at early stages of development and muscular hypotonia, weak deep tendon reflexes, and delayed motor milestones are unspecific neurological signs. However, a particular gestalt may help to identify genetic diseases, and, at the present time, new technologies of facial pattern recognition have demonstrated their usefulness in a wide number of genetic conditions [14]. 

The cerebellum expresses a variety of different functions in addition to motor action, including affective skills, working memory, response timing and attentional control, and many of the patients reported on in the literature show intellectual disability [15]. Moreover, the cerebellum is strongly associated with autism spectrum disorder (ASD); pathological studies have found consistent abnormalities in Purkinje cells in ASD patients; in addition, the cerebellum is involved in associative learning, cognition, and emotional function, which are altered in ASD [16]. Two of our patients show intellectual disability, and the most severely affected patient (patient 1) also shows autistics traits. The younger patients have ADHD with remarkable good response to methylphenidate and guanfacine, which might be explained by the impairment in cognitive executive functions and working memory described in cerebellar disorders [15].

The three patients showed an improvement in all neurodevelopmental areas, despite two of them showing a progressive cerebellar atrophy in sequential MRI. The most severe patient shows the greatest cerebellar atrophy, which suggests that there might be a correlation between the neurological impairment (including motor, speech and cognitive skills) and the degree of cerebellar atrophy (Figure 1).

Acetazolamide therapy showed clear benefit in patient 2, but not in patient 3 carrying the same Ca_V_2.1 amino acid substitution; however, the development of renal calculi prompted us to stop the therapy. In patient 1, parents perceived a benefit in muscle tone and communication intention after acetazolamide treatment, but the lack of specific scales to evaluate cerebellar syndrome before and after the treatment limits our conclusions. 

**Figure 4:**
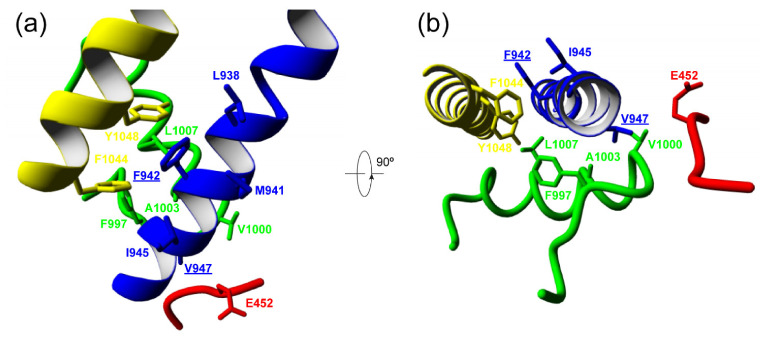
Internal Ca_V_1.1 channel amino acid interactions affected by mutations F942L and V947M. Structure of the rabbit Ca_V_1.1 channel showing the side-chains of F942 and V947 and Yasara-identifed interacting residues from side (**a**) and top (**b**) views. Residues L938 and M941 are hidden in panel B for visualization purposes. DII S1-S2 loop and DIII S5, P-loop and S6 are colored in red, blue, green and yellow, respectively.

Knowledge of the mutation’s effect on protein function is important to improve our understanding of the phenotype of patients with *CACNA1A*-related disease and to contribute to the development of new treatment approaches. In this respect, no functional studies are available for the previously unreported F1394L variant. Still, the affected residue is located in a region of the S5 segment at DIII contributing to the channel pore. This region includes invariant amino acids conserved in the rabbit Ca_V_1.1 (Figure 2), in Ca_V_2.1 through evolution (Figure 3a, left) and in all human Ca_V_ channels (Figure 3b, left), which suggests high functional relevance. In this sense, two other amino acid substitutions in the same S5 pore segment where F1394L is located, Y1385C (corresponding to Y1387C in α_1A_ isoform 2 and Y1384C in isoform 3) and V1396M (corresponding to V1399M in α_1A_ isoform 2), have been reported in association with congenital ataxia with cerebellar atrophy, psychomotor delay, intellectual disability, epilepsy (including different kinds of refractory generalized seizures), HM and acute coma (Figure 3, left) [17,18,19]. For mutation V1396M/V1399M (five residues upstream of F1394L in α_1A_ isoform 2), the functional analysis of the heterologously expressed murine Ca_V_2.1 ortholog carrying the equivalent variant (V1347M) (Figure 3a, left) shows gain-of-function effects due to higher current density and lower voltage threshold for channel activation, when compared to the wild-type (WT) channel [18]. Such increase in channel activity is consistent with the close proximity of the affected residue to the initial pore-loop region that contributes to both the entrance of Ca^2+^ into the selectivity filter vestibule and into the docking site for the regulatory α_2_δ subunit [12], which is essential for the proper functional expression of the pore-forming α_1A_ subunit at the plasma membrane [3]. Residues V1396/V1399 and F1394 are in fact fully conserved in the pore-forming α_1S_ subunit of the rabbit Ca_V_1.1 channel for which structural data are available [12], where they correspond to residues V947 and F942, respectively (Figure 2b). Therefore, we can evaluate whether the changes of these amino acids to methionine and leucine, respectively, and linked to cerebellar dysfunction may modify structural features of the channel subunit. According to Yasara calculation of amino acid interactions on the rabbit Ca_V_1.1 structure, V947 mainly stablish hydrophobic contacts with three residues at the P-loop of domain III (F997, V1000 and A1003) and with the amino acid E452 at the extracellular loop between S1 and S2 of domain II (Figure 4 and Table 2 top). All these residues are conserved or semiconserved in human Ca_V_2.1, except E452 (the equivalent amino acid is a valine) (see Table 2 Legend). By using the Amino Acid Interactions (INTAA) web server, we also estimated pairwise interaction energy (kJ/mol) between the side-chain of V947 and its neighbor residues and found that mutation V947M worsen all hydrophobic interactions, in particular with E452 (even when this amino acid is substituted by a valine as found in human Ca_V_2.1). This may alter the correct conformation of the voltage sensor, thus explaining the increase in voltage sensitivity reported for the VM mutant channel [18].

In the case of F942, Yasara identifies the interactions with other residues located at regions delineating the channel pore in domain III: L938, M941 and I945 at the S5 segment, L1007 at the P-loop, and F1044 and Y1048 at the distal half of S6 segment that faces the cytosolic side and line the inner pore vestibule of the channel (Figure 4). Again, all these interactions, specially between F942 and Y1048 (corresponding to F1394 and Y1497 in Ca_V_2.1), are expected to be energetically worse by mutation F942L (Table 2), in this case caused by the loss of the aromatic ring and consequently the interaction. This may prevent the proper 3D arrangement of the channel inner pore. Although further research, including electrophysiological analysis, is required to confirm this hypothesis, interestingly, a gain-of-function Ca_V_2.1 genetic variant in that precise S6 pore area (ΔF1502) associated to congenital ataxia and hemiplegic migraine, has been reported to improve voltage-dependent gating of Ca_V_2.1 by strongly decreasing the voltage threshold for channel opening, fastening activation kinetics, and slowing down both the deactivation and the inactivation of the channel [13,20].

The functional consequences of a third mutation at the S5 segment of Ca_V_2.1 domain III, Y1385C, has been recently reported and both gain- and loss-of-function effects have been observed [21]. As found for V1396M, Y1385C also favors channel opening by voltage (gain-of-function) [21]. However, Y1385C has the contrary effect on Ca_V_2.1 current density, which is strongly reduced by the mutation without alteration of channel trafficking to the cell membrane (loss-of-function) [21]. Besides, Y1385C also reduces the voltage threshold for channel inactivation (loss-of-function). Nevertheless, the combination of all these alterations results in a global gain-of-function due to higher persistent activity when compared to WT channels, which can lead to a greater Ca^2+^ influx into cells over a physiologically relevant window of membrane potentials near the resting potential [21]. This is consistent with the association of Y1385C to HM, which is produced by *CACNA1A* gain-of-function mutations [1].

In the same F1394 position, a single nucleotide deletion (c.4182delC) that resulted in a putative truncated protein due to premature termination, was found in several members of an EA2 family showing variation of clinical symptoms among the carriers, ranging from severe early-onset (at the age of 1-2) weekly episodes to less severe phenotype with first attacks occurring in childhood and prolonged attack-free periods [22,23]. As all Ca_V_2.1 truncations, it is expected to produce channel loss-of-function that is the cause of the majority of EA2 cases [1].

Several mutations affecting residues located at other Ca_V_2.1 S5 segments have also been linked to neurological disorders, including episodic ataxia type 2 (EA2) and HM (Figure 3c, left). Two mutations affecting the Y248 residue at the S5 helix of domain I (DI) (Y248C and Y248N) and mutation L621R at the S5 helix of domain II (DII) were found in association with EA2 [24,25], a disease mostly due to Ca_V_2.1 null or reduced activity [1]. Only mutation L621R has been studied at the functional level after heterologous expression. Unfortunately, no significant effect on Ca_V_2.1 channel function was found to corroborate the expected loss-of-function effect. Thus, L621R did not alter Ca_V_2.1 current density, nor channel rate of activation or inactivation [26]. Among the mutations linked to HM (mainly produced by Ca_V_2.1 gain-of-function) are G230V at S5-DI [27], V1695I (referred to α_1A_ isoform 3 and corresponding to V1700I in α_1A_ isoform 2) [28] and I1710T (also referred to as I1709T and corresponding to I1714T in α_1A_ isoform 2) [29], both at S5-DIV. Functional study of the heterologously expressed murine Ca_V_2.1 ortholog carrying the equivalent G230V mutation (G232V) suggests a loss-of-function effect due to reduced channel expression at the cell surface [18]. Whether this effect occurs only after heterologous expression in mammalian cells but not in patients’ neurons remains controversial. Indeed, most HM-linked *CACNA1A* mutations show decreased density of functional Ca_V_2.1 channels in the plasma membrane depending on the cell expression system [1], making it difficult to evaluate both the consequences of this effect in vivo and its relevance for the associated clinical phenotype, if any. In contrast, functional analysis of mutation V1695I/V1700I reveals increased channel activity due to reduced voltage threshold for Ca_V_2.1 activation (by ~4 mV), slowed channel inactivation, and lessened direct G protein-mediated inhibition [30,31].

Regarding the R1664Q/R1669Q mutation affecting the second gating charge (R2) at DIV S4 voltage-sensing helix, it has been previously found to be linked to different ataxic phenotypes, such as EA2 and congenital ataxia [32,33,34]. In some cases, carriers present cerebellar atrophy and other symptoms, such as psychomotor delay, intellectual disability, and migraine without focal deficits. Studies performed in transgenic *Drosophila* flies show that the introduction of the R1664Q/R1669Q variant in the equivalent Ca_V_2.1 (cacophony calcium-channel) does not allow rescue of synaptic transmission in a Ca_V_2.1-deficient background, as happens with the WT channel [33]. This suggests that R1664Q/R1669Q is a loss-of-function variant, which is consistent with its linkage to EA2, mainly produced by *CACNA1A* loss-of-function mutations.

Other mutations of clinical relevance also modify R2 residues, as occurs with the R1664Q/R1669Q variant. This is the case with R198Q, linked to EA2 and affecting the R2 gating charge at the DI S4 segment, (Figure 3c, right), and a gain-of-function mutation linked to congenital ataxia, psychomotor delay, intellectual disability, febrile seizures, and HM (R1350Q, named as R1349Q by some authors and corresponding to R1352Q in α_1A_ isoform 2) that locates at the equivalent R2 gating charge of DIII S4 helix (Figure 3c, right) [4,35,36,37,38].

## 4. Materials and Methods

Patients with a molecular confirmation of de novo variants in *CACNA1A*, attended at the Neurology Department of Hospital Sant Joan de Déu, and presenting with early onset ataxia were eligible. Evaluations from November 2017 to November 2020 were included. Patients with *CACNA1A* variants showing early epileptic encephalopathy or those with familial antecedents of HM were excluded. 

For measuring cerebellar syndrome, neurological exam and assessment through ICARS, recently been validated in children with cerebellar syndrome, were performed [39]. Regarding dysmorphic evaluation, patients’ measures were compared with age and gender-related reference values from the Hall’s Handbook of Normal Physical Measurements [40].

Brain MRI exams included T1- and T2-weighted, diffusion-weighted and FLAIR sequences. To evaluate MRI cerebellar images, 2D analysis was performed using the midsagittal vermis relative diameter (MVRD) already validated in children with cerebellar atrophy [41]. This is calculated using a midsagittal section and measuring total posterior cranial fossa diameter in a linear segment from the posterior commissure to the opisthion and the largest sagittal diameter of the cerebellum parallel to the previous linear segment. The MVRD was compared to two sex- and age-matched controls from a historical cohort [41]. Lower indices denote greater cerebellar atrophy. 

For molecular studies genomic DNA was isolated from venous whole blood. Mutational analysis was performed by genomic DNA analysis both in patient’ and parent samples. In the three patients a targeted gene panel of ataxia-causing genes was run. The identified likely pathogenic heterozygous variants were confirmed by Sanger sequencing in the proband and the parents, ratifying a de novo condition. 

For analysis of interaction energies, calculations were performed using the rabbit Ca_V_1.1 model (PDB id: 5gjv) and the F942L and V947M mutants, which were generated using Chimera’s rotamers tool [42]. We used Yasara’s algorithm [43] to minimize energy of models and identify interactions between residues of interest. The INTAA web server [44], which uses Lennard-Jones potential and point charges electrostatics, was used to calculate pairwise side chain interaction energies.

## 5. Conclusions

In conclusion, our results suggest that among the broad spectrum of *CACNA1A*-related phenotypes, non-progressive congenital ataxia is associated with cognitive impairment and dysmorphic features, constituting a recognizable syndromic neurodevelopmental disorder. Further studies in larger series of patients are needed to establish whether the recognition of this pattern in patients with nonspecific motor and/or cognitive impairment and cerebellar atrophy might aid in early diagnosis of *CACNA1A*-related disease, thereby allowing a targeted management and care of the patients and their families. Deepening of our knowledge of the effect of each mutation on protein function, to distinguish between both gain- and loss-of-function, is required to advance in the development of new personalized therapies.

## Figures and Tables

**Table 1:** GA: Gestational age; HELLP syndrome: hemolysis, elevated liver enzymes, low platelet count, related with pregnancy; SD: standard deviation; OFC: occipito-frontal circumference; WIPPSI: Wechsler Preschool and Primary Scale of Intelligence; ADHD: attention deficit and hyperactivity disorder; MPD: Methyphenidate; MVRD: Midsaggital vermis relative diameter. * Subscores: Language 77, socialization 80, coordination 75, postural 34.

Patient/Gender	1 Female	2 Male	3 Female
Age	8 years	20 years	7 years
Molecular findings/ inheritance	c.4182C>A p.Phe1394Leu/De novo	c.4991G>A p.Arg1664Gln/De novo	c.5006G>A p.Arg1669Gln/De novo
Pregnancy & delivery/GA	Twin pregnancy/HELLP syndrome/C-section/ 35 weeks	Normal/eutocic/41 weeks	Normal pregnancy/forceps/40 + 6 weeks
Somatometry at birth (SD)	Weight 2150 g (+1.0SD) Height 43.5cm (+0.7 SD) OFC 32 cm (+0.5 SD)	Weight 3520 g (+0,1 SD) Height 51 cm (+0.1 SD) OFC 36 cm (+0.4 SD)	Weight 3400 g (+0.3 SD) Height 51 cm (+0.6 SD) OFC 35 cm (+0.1 SD)
Somatometry at last evaluation (SD)	19.5 kg (−1.5 SD) 1.16 m (−0.5 SD) OFC 52.5 cm (+0.5 SD)	77.2 kg (+0.2 SD) 176 m (−0.2 SD) OFC 59 cm (+1.5 SD)	22.6 kg (−0.89 9 SD) 1.23 m (−0.69 7 SD) OFC 53.5 cm (+1.2 SD)
Initial neurological symptoms	Hypotonia Severe developmental delay	Hypotonia Developmental delay	Hypotonia Development delay
Cerebellar syndrome	Truncal ataxia and stereotypes Strabismus, terminal nystagmus	Mild ataxia/Dysarthria Oculomotor apraxia Nystagmus	Mild ataxia
Neurodevelopment	Sitting position: 27 months Walk only with stroller: 5 years No speech (guttural sounds) Special schooling	Sitting position: 8 months Independent walking: 30 months Language delay Occupational school	Sitting position: 9 months Independent walking: 30 months Language Delay Ordinary school with support
Other neurological symptoms	Intellectual disability Autistic traits Attention deficit (treated with guanfacine)	Mild intellectual disability Uncontrolled lateral head movements without consciousness abnormalities.	Mild intellectual disability Brunet−Lézine (30 months) 67 * WIPPSI IV (5.5 years): verbal IQ 70, performance IQ 68. ADHD (MPD)
Cranial magnetic resonance	6 months: ventricular enlargement and increased extra−axial spaces. Normal posterior fossa structures. 16 months: cerebellar atrophy: increased interfolia spaces mainly in vermis. MVRD = 0.66 (mean for controls 0.77). 4 years 10 months: progression of generalized cerebellar atrophy. MVRD = 0.51 (mean for controls 0.80)	6 years 10 months: cerebellar atrophy, with a MVRD = 0.76 (mean for controls 0. 82). 13 years 2 months: progressive generalized cerebellar atrophy. MVRD = 0.64 (mean for controls 0.87)	15 months: no signs of cerebellar atrophy. 24 months: cerebellar atrophy: increase of interfolia spaces in the superior vermis
ICARS assessment	No collaboration, no comprehension	17 years: 14/100 19 years: 12/100	4 years: 28/100 6 years: 19/100
Acetazolamide therapy(at least during 8 months)	12 mg/kg/day in two doses Improvement in muscle tone and communication intention. No objective positive response in the long term.	250 mg /12 h Improvement in motor symptoms. Abolished stereotyped episodes. Withdrawn due to lithiasis	12 mg/kg/day in two doses. No objective positive response

**Table 2:** Calculation of pairwise interaction energy (kJ/mol) between the side-chains of residues V947 (top) or F942 (bottom) (and their corresponding methionine and leucine mutants, respectively) and Yasara-calculated interacting residues. The values have been obtained with the Amino Acid Interactions (INTAA) web server using the WT or mutant rabbit Ca_V_1.1 models, as indicated (PDB id: 5gjv). All residues interacting with V947 and F942 are conserved or semiconserved in human Ca_V_2.1, except E452. Thus, V947 corresponds to V1396, F997 to Y1443, V1000 to V1446, A1003 to A1449 and E452 to V507 (the numbering of these Ca_V_2.1 residues are referred to isoform 3). In the same way, F942 corresponds to F1394, L938 to F1390, M941 to I1393, I945 to V1397, L1007 to L1456, F1044 to F1493 and Y1048 to Y1497 (in this case, the numbering of the Ca_V_2.1 amino acids are referred to isoform 2). As residue E452 is not conserved in human Ca_V_2.1 (where the corresponding residue is a valine), we also evaluated how V947M mutation could affect the energy interaction with a valine at the same position (V452) by exchanging the sequence of the DII loop S1–S2 of rabbit Ca_V_1.1 for that of human Ca_V_2.1.

	**E452**	**V452**	**F997**	**V1000**	**A1003**	
**V947**	−3.98	−3.96	−2.02	−3.06	−2.08	
**M947**	47.27	30.3	−1.09	−1.93	−0.67	
**Δ**	+51.25	+34.26	+0.93	+1.13	+1.41	
	**L938**	**M941**	**I945**	**L1007**	**F1044**	**Y1048**
**F942**	−5.54	−8.22	−4.72	−2.96	−2.34	−9.41
**L942**	−3.74	−3.30	−3.71	−2.94	7.26	37.47
**Δ**	+1.8	+4.92	+1.01	+0.02	+9.6	+46.88

## Data Availability

The data presented in this study are available on request from the corresponding author. The data are not publicly available due to protection data laws protecting personal medical information.

## Supplementary Materials

The following are available online at https://www.mdpi.com/article/10.3390/ijms22105180/s1, Table S1: Previously reported CACNA1A variants linked to congenital ataxia (CA) including functional consequences and molecular localization (protein domain).
