# Supplementary material for: CACNA1A Mutations Causing Early Onset Ataxia: Profiling Clinical, Dysmorphic and Structural-Functional Findings
Source: Int J Mol Sci. 2021 May 13;22(10):5180. doi: 10.3390/ijms22105180 (PMC8153625; doi:10.3390/ijms22105180)
Supplement: Supplementary file 1 [file ijms-22-05180-s001.zip › ijms-1202093-supplementary.pdf]

Supplementary Table

Previously reported *CACNA1A* variants linked to congenital ataxia (CA) including functional consequences and molecular localization (protein domain). Adapted from Izquierdo-Serra et al [1].

	**Variant (p.)**	**Domain**	**References (clinical descriptions of CA linked to variants in literature)**
**Gain of Function (GoF)**	T666M	Pore-Selectivity filter	[2,3]
	D715E	Pore	[3,4]
	V1396M	Pore	[5,6]
	ΔF1502	Pore	[7,8]
	I1811L	Pore	[9]
	R1349Q/R1350Q/R1352Q	Voltage sensor	[5, 10-13]
	S218L	Linker S4-S5	[14,15]
**Loss of function (LoF)**	R1664Q	Voltage sensor	[16, Present work]
**GoF and LoF**	R1673P	Voltage sensor	[17]
	Y1384C/Y1385C	Pore	[18, 19]
**Not known**	I712V	Pore	[20]
	V1696F	Pore	[21]
	S1799L	Pore	[22]
	F1394L	Pore	[Present work]
	I1338T	Voltage sensor	[23]
	I1342T	Voltage sensor	[5]
	R1359W	Voltage sensor	[22]

Supplementary Table References:

1. Izquierdo-Serra, M.; Fernández-Fernández, J.M.; Serrano, M. Rare CACNA1A mutations leading to congenital ataxia. *Pflugers Arch* **2020**,,472, 791-809. doi:10.1007/s00424-020-02396-z
2. Kors, E.E.; Haan, J.; Giffin, N.J.; Pazdera, L.; Schnittger, C.; Lennox, G. G.; Terwindt, G. M.; Vermeulen, F. L.; Van den Maagdenberg, A. M.; Frants, R. R.; Ferrari, M. D. Expanding the phenotypic spectrum of the CACNA1A gene T666M mutation: a description of 5 families with familial hemiplegic migraine. *Arch Neurol* **2003**, 60, 684-8. doi:10.1001/archneur.60.5.684
3. Ducros, A.; Denier, C.; Joutel, A.; Vahedi, K.; Michel, A.; Darcel, F.; Madigand, M.; Guerouaou, D.; Tison, F.; Julien, J.; Hirsch, E.; Chedru, F.; Bisgård, C.; Lucotte, G.; Després, P.; Billard, C.; Barthez, M. A.; Ponsot, G.; Bousser, M. G.; Tournier-Lasserve, E. Recurrence of the T666M calcium channel CACNA1A gene mutation in familial

hemiplegic migraine with progressive cerebellar ataxia. *Am J Hum Genet* **1999**, 64, 89-98. doi:10.1086/302192

4. Ducros, A.; Denier, C.; Joutel, A.; Cecillon, M.; Lescoat, C.; Vahedi, K.; Darcel, F.; Vicaut, E.; Bousser, M.G.; Tournier-Lasserve, E. The clinical spectrum of familial hemiplegic migraine associated with mutations in a neuronal calcium channel. *N Engl J Med* **2001**, *345*, 17–24.
5. Travaglini, L.; Nardella, M.; Bellacchio, E.; D'Amico, A.; Capuano, A.; Frusciante, R.; Di Capua, M.; Cusmai, R.; Barresi, S.; Morlino, S.; Fernández-Fernández, J.M.; Trivisano, M.; Specchio, N.; Valeriani, M.; Vigevano, F.; Bertini, E.; Zanni, G. Missense mutations of CACNA1A are a frequent cause of autosomal dominant nonprogressive congenital ataxia. *Eur J Paediatr Neurol* **2017**, *21*, 450–6.
6. Jiang, X.; Raju, P.K.; D'Avanzo, N.; Lachance, M.; Pepin, J.; Dubeau, F.; Mitchell, W.G.; Bello-Espinosa, L.E.; Pierson, T.M.; Minassian, B.A.; Lacaille J.C.; Rossignol, E. Both gain-of-function and loss-of-function de novo CACNA1A mutations cause severe developmental epileptic encephalopathies in the spectrum of Lennox-Gastaut syndrome. *Epilepsia* **2019**, *60*, 1881–94.
7. Bahamonde, M. I.; Serra, S. A.; Drechsel, O.; Rahman, R.; Marcé-Grau, A.; Prieto, M.; Ossowski, S.; Macaya, A.; Fernández-Fernández, J. M. A Single Amino Acid Deletion (ΔF1502) in the S6 Segment of CaV2.1 Domain III Associated with Congenital Ataxia Increases Channel Activity and Promotes Ca2+ Influx. *PLoS One* **2015**, 10, e0146035. Published 2015 Dec 30. doi:10.1371/journal.pone.0146035
8. García-Segarra, N.; Gautschi, I.; Mittaz-Crettol, L.; Kallay Zetchi, C.; Al-Qusairi, L.; Van Bemmelen, M.X.; Maeder, P.; Bonafé, L.; Schild, L.; Roulet-Perez, E. Congenital ataxia and hemiplegic migraine with cerebral edema associated with a novel gain of function mutation in the calcium channel CACNA1A. *J Neurol Sci* **2014**, *342*, 69–78.
9. Terwindt, G. M.; Ophoff, R. A.; Haan, J.; Vergouwe, M. N.; van Eijk, R.; Frants, R. R.; Ferrari, M. D. Variable clinical expression of mutations in the P/Q-type calcium channel gene in familial hemiplegic migraine. Dutch Migraine Genetics Research Group. *Neurology* **1998**, 50,1105-10. doi:10.1212/wnl.50.4.1105
10. Tantsis, E.M.; Gill, D.; Griffiths, L.; Gupta, S.; Lawson, J.; Maksemous, N.; Ouvrier, R.; Riant, F.; Smith, R.; Troedson, C.; Webster, R.; Menezes, M.P. Eye movement disorders are an early manifestation of CACNA1A mutations in children. *Dev Med Child Neurol* **2016**, *58*, 639–44.
11. Blumkin, L.; Leshinsky-Silver, E.; Michelson, M.; Zerem, A.; Kivity, S.; Lev, D.; Lerman-Sagie, T. Paroxysmal tonic upward gaze as a presentation of de-novo mutations in CACNA1A. *Eur J Paediatr Neurol* **2015**, *19*, 292–7.
12. Blumkin, L.; Michelson, M.; Leshinsky-Silver, E.; Kivity, S.; Lev, D.; Lerman-Sagie, T. Congenital ataxia, mental retardation, and dyskinesia associated with a novel CACNA1A mutation. *J Child Neurol* **2010**, *25*, 892–7.
13. Zhang, L.; Wen, Y.; Zhang, Q.; Chen, Y.; Wang, J.; Shi, K.; Du, L.; Bao, X. *CACNA1A* Gene Variants in Eight Chinese Patients With a Wide Range of Phenotypes. *Front Pediatr* **2020**, 8, 577544. Published **2020** Dec 3. doi:10.3389/fped.2020.577544
14. Stam, A. H.; Luijckx, G. J.; Poll-Thé, B. T.; Ginjaar, I. B.; Frants, R. R.; Haan, J.; Ferrari, M. D.; Terwindt, G. M.; van den Maagdenberg, A. M.. Early seizures and cerebral oedema after trivial head trauma associated with the CACNA1A S218L mutation. *J Neurol Neurosurg Psychiatry* **2009,** 80, 1125-9. doi:10.1136/jnnp.2009.177279Dzfbzgfb

15. Tonelli, A.; D'Angelo, M.G.; Salati, R.; Villa, L.; Germinasi, C.; Frattini, T.; Meola, G.; Turconi, A.C.; Bresolin, N.; Bassi, M.T. Early onset, non fluctuating spinocerebellar ataxia and a novel missense mutation in CACNA1A gene. *J Neurol Sci* **2006**, *241*, 13–7.
16. Tyagi, S.; Bendrick, T.R.; Filipova, D.; Papadopoulos, S.; Bannister, R.A. A mutation in Cav2.1 linked to a severe neurodevelopmental disorder impairs channel gating. *J Gen Physiol* **2019**,151, 850-9. doi:10.1085/jgp.201812237
17. Vahedi, K.; Denier, C.; Ducros, A.; Bousson, V.; Levy, C.; Chabriat, H.; Haguenau, M.; Tournier-Lasserve, E.; Bousser, M. G. CACNA1A gene de novo mutation causing hemiplegic migraine, coma, and cerebellar atrophy. *Neurology* **2000**, 55, 1040-2. doi:10.1212/wnl.55.7.1040
18. Gandini, M.A.; Souza, I.A.; Ferron, L.; Innes, A.M.; Zamponi, G.W. The de novo CACNA1A pathogenic variant Y1384C associated with hemiplegic migraine, early onset cerebellar atrophy and developmental delay leads to a loss of Cav2.1 channel function. *Mol Brain* **2021**, 14, 27. Published 2021 Feb 8. doi:10.1186/s13041-021-00745-2
19. Guerin, A.A., Feigenbaum, A.; Donner, E.J.; Yoon, G. Stepwise developmental regression associated with novel CACNA1A mutation. *Pediatr Neurol* **2008**, 39, 363-4. doi:10.1016/j.pediatrneurol.2008.07.030
20. Yamazaki, S.; Ikeno, K.; Abe, T.; Tohyama, J.; Adachi, Y. Hemiconvulsion-hemiplegia-epilepsy syndrome associated with CACNA1A S218L mutation. *Pediatr Neurol* **2011**, 45, 193-6. doi:10.1016/j.pediatrneurol.2011.04.010
21. de Vries, B.; Stam, A. H.; Beker, F.; van den Maagdenberg, A. M.; Vanmolkot, K. R.; Laan, L.; Ginjaar, I. B.; Frants, R. R.; Lauffer, H., Haan, J.; Haas, J. P.; Terwindt, G. M.; Ferrari, M. D. CACNA1A mutation linking hemiplegic migraine and alternating hemiplegia of childhood. *Cephalalgia* **2008**, 28, 887-91. doi:10.1111/j.1468-2982.2008.01596.x
22. Ohba, C.; Osaka, H.; Iai, M.; Yamashita, S.; Suzuki, Y.; Aida, N.; Shimozawa, N.; Takamura, A.; Doi, H.; Tomita-Katsumoto, A.; Nishiyama, K.; Tsurusaki, Y.; Nakashima, M.; Miyake, N.; Eto, Y.; Tanaka, F.; Matsumoto, N.; Saitsu, H. Diagnostic utility of whole exome sequencing in patients showing cerebellar and/or vermis atrophy in childhood. *Neurogenetics* **2013**, 14, 225-32. doi:10.1007/s10048-013-0375-8
23. Frusciante, R.; Capuano, A.; Travaglini, L.; Zanni, G.; Vigevano, F.; Bertini, E.; Valeriani, M. P016. Congenital ataxia, hemiplegic migraine due to a novel mutation of CACNA1A: a case report. *J Headache Pain* **2015**, 16, A146. doi:10.1186/1129-2377-16-S1-A146
